# If you build it, they still may not come: outcomes and process of implementing a community-based integrated knowledge translation mapping innovation

**DOI:** 10.1186/1748-5908-5-47

**Published:** 2010-06-16

**Authors:** S Michelle Driedger, Anita Kothari, Ian D Graham, Elizabeth Cooper, Eric J Crighton, Melanie Zahab, Jason Morrison, Michael Sawada

**Affiliations:** 1 Department of Community Health Sciences, University of Manitoba, S113-750 Bannatyne Ave, Winnipeg, Manitoba, R3E 0W3, Canada; 2 Faculty of Health Sciences, Bachelor of Health Sciences, University of Western Ontario, Arthur and Sonia Labatt Health Sciences Building, Room 222, London, Ontario, N6A 5B9, Canada; 3 VP Knowledge Translation, Canadian Institutes of Health Research, 160 Elgin Street, 9th Floor, Address Locator 4809A, Ottawa, ON, K1A 0W9, Canada; 4 Department of Geography, University of Ottawa, Ottawa ON, K1N 6N5, Canada; 5 Department of Biosystems Engineering, University of Manitoba, E2-376 Engineering Building , University of Manitoba, Winnipeg MB, R3T 5V6, Canada; 6 Laboratory for Applied Geomatics and GIS Science (LAGGISS), Department of Geography, University of Ottawa, Ottawa ON, K1N 6N5, Canada

## Abstract

**Background:**

Maps and mapping tools through geographic information systems (GIS) are highly valuable for turning data into useful information that can help inform decision-making and knowledge translation (KT) activities. However, there are several challenges involved in incorporating GIS applications into the decision-making process. We highlight the challenges and opportunities encountered in implementing a mapping innovation as a KT strategy within the non-profit (public) health sector, reflecting on the processes and outcomes related to our KT innovations.

**Methods:**

A case study design, whereby the case is defined as the data analyst and manager dyad (a two-person team) in selected Ontario Early Year Centres (OEYCs), was used. Working with these paired individuals, we provided a series of interventions followed by one-on-one visits to ensure that our interventions were individually tailored to personal and local decision-making needs. Data analysis was conducted through a variety of qualitative assessments, including field notes, interview data, and maps created by participants. Data collection and data analysis have been guided by the Ottawa Model of Research Use (OMRU) conceptual framework.

**Results:**

Despite our efforts to remove all barriers associated with our KT innovation (maps), our results demonstrate that both individual level and systemic barriers pose significant challenges for participants. While we cannot claim a causal association between our project and increased mapping by participants, participants did report a moderate increase in the use of maps in their organization. Specifically, maps were being used in decision-making forums as a way to allocate resources, confirm tacit knowledge about community needs, make financially-sensitive decisions more transparent, evaluate programs, and work with community partners.

**Conclusions:**

This project highlights the role that maps can play and the importance of communicating the importance of maps as a decision support tool. Further, it represents an integrated knowledge project in the community setting, calling to question the applicability of traditional KT approaches when community values, minimal resources, and partners play a large role in decision making. The study also takes a unique perspective--where research producers and users work as dyad-pairs in the same organization--that has been under-explored to date in KT studies.

## Background

It is well-recognized in the academic literature and in practice that research utilization takes considerable time and is marked by inconsistencies across different users and organizations [1]. Recent efforts focus on trying to support an interactive exchange between researchers and research users [2,3], a participatory process referred to by the Canadian Institutes of Health Research [4] as integrated knowledge translation (KT). Most KT activities have identified the research user as a health practitioner, administrator, or policymaker, and desired outcomes involve changes in knowledge, attitudes, behaviours, programs, or policies [5]. The underlying assumptions of contemporary perspectives of KT suggest that the producer and user of research reside, metaphorically speaking, in 'two (separate) communities' [6,7]. We begin, however, from the position that many research producer/user pairs--or what we refer to as dyads--work in close proximity, and represent an understudied dimension of KT. In government, policy analysts evaluate and summarize policy options and research for senior bureaucrats who make decisions. At a more local level, public health unit managers apply research provided by in-house epidemiologists. In this project, the dyads of interest are data analysts and their managers working in early childhood development centres called Ontario Early Years Centres (OEYCs). This dyad situation, where local data are generated within organizations, has yet to be considered in the KT literature.

OEYCs are part of a Canadian federal/provincial/territorial early child development strategy with the mandate to provide services to parents/caregivers with children under the age of six [8]. The goal of these programs and services is to help improve a child's readiness to learn when they become school-aged, as measured through an early development instrument (EDI). The EDI is composed of a population-based questionnaire, collected across Canada. In Ontario, the Ontario Early Years program began in 2002 with 15 pilot sites, and now represents 103 communities. The OEYCs consist of data analysts who are stewards of Early Years' data. These analysts are a 'valuable resource' to the communities they serve, and a 'clearing house' for information on Early Years in their community [9]. The EDI is one of the primary datasets used by OEYCs in program planning and decision making, as well as for community based outreach. Other data sources used by OEYCs include: census data, locally collected data from community program and evaluation surveys, and locally relevant data from health units and schools. Most of these datasets can be geo-referenced (often via postal codes) for mapping purposes. Thus, an opportunity to use local data in decision making, and further, to explore the role that maps as a KT tool might play in this process, presented itself. What makes this KT context unique is that the research producers (the OEYC data analysts), and the users (their managers) reside in the same community-based organization.

This paper presents the results of the second phase of a two-phase project. The project's central research question asks: to what extent can mapping software and maps support evidence-based decision making about program planning and policies in OEYCs? Phase one involved a participatory design process to develop a web-based mapping software (EYEMAP) tailored to the needs of data analysts (see [10]), as well an assessment of the modifiable and non-modifiable factors that needed to be addressed to encourage the adoption of maps as a KT tool (see [11]). Findings demonstrated that we needed to provide adequate training to our potential adopters in making and interpreting maps, address their general perceptions and attitudes towards maps and mapping, and ensure that a common terminology was familiar to both data analysts and managers so that managers would know the types of spatial questions that could be asked of data analysts to support decisions based on available data sources. In order to address these barriers, which are frequently encountered in other information system uptakes [12], phase two of the project involved providing a series of four tailored interventions to our KT dyads. We paid particular attention to providing adequate training in the classification of spatial data (*i.e.*, knowing when one classification system is preferable over another depending on the type of data used) and best practices in mapping. In addition to the above barriers assessment, to help facilitate success, we conducted a short telephone interview with participants prior to the third intervention to further assess participant progress and individual training needs. Our project was collaborative and participatory, in that we sought to involve our project participants throughout the research process, to ensure that our interventions were tailored to meet their needs. Following these interventions, the purpose of this article is to evaluate the use and impacts of mapping software and maps by OEYC data analysts and managers, respectively. A critical discussion on the process of 'doing integrated KT' is also presented.

## Methods

The Ottawa Model for Research Use (OMRU) [13-16] guided data collection and analysis. The OMRU is an interactive planned-action theory in that change in target behaviour is engineered as opposed to something that emerges haphazardly. The OMRU assembles diverse aspects of the process of healthcare services research use into a simple but widely applicable framework for assessing barriers and facilitators to utilization. In the OMRU, the utilization of research is dependent on three sources: the innovation, the potential users, and the environment. Potential users' perceptions of the attributes, or characteristics of the innovation, can influence their decisions to use the innovation in either positive or negative ways. Potential users of maps (the KT dyads)--the producer (data analyst) and user (manager)--have particular knowledge, attitudes, skills, and motivations that may affect uptake, but, motivation, basic skills, and access to technology still may not ensure that the tools may be fully utilized [12]. The environment also contains structural and social influences that may foster or impede the uptake of an innovation. The strength of OMRU is its prescriptive feature--assessing, monitoring, and evaluating--throughout the process to ensure that interventions are appropriately tailored to meet the needs of potential users.

Carol Weiss has described ways in which the utilization of research can be conceptualized [17,18]. The most direct way is for research to be used instrumentally, where there is tangible evidence of its influence. In this study, maps might be used instrumentally if they are cited in organizational documents (*e.g.*, annual reports) or referred to in meeting minutes during decisions about childhood programs. Research can also serve an enlightenment function, which is more difficult to ascertain because it involves shifting the way that a research user perceives a social problem; further, it can take time for the research to influence the user's conceptual understanding of the issue. For example, users of maps may, over time, be increasingly capable of articulating the importance of using maps to display community-based data. Weiss describes a third way in which research might be used: symbolically, or to support a decision that has already been made [18]. This might be observed in the current study if managers state that they made a program or policy decision, and then found that their decision was subsequently reinforced by the data displayed in a map generated by a data analyst.

### Participant sample

We purposively sampled OEYCs who were part of an earlier mapping project to further encourage research partnerships. While the invited OEYCs participated, due to staff turnover, none of the original data analysts were available. Other OEYCs in Southern Ontario were also invited to participate. Because our web-based mapping software was housed on a secure server, the number of participants had to be limited to what could be functionally supported by the hardware, thereby avoiding a potential intervention uptake barrier. At the start of the project, nine manager-data analyst pairs agreed to participate in the study.

### Description of KT intervention

The specific nature and content of the KT intervention was refined based on an assessment of each group's needs, and designed to provide external facilitation [19,20]--training/education, troubleshooting support, and providing technical (software) and other mapping advice (principles and practice of GIS). This was done through extensive preliminary interviews to determine: types of GIS software used other than the web-based software (EYEMAP) developed by the project (as per phase one); types of data collected (spatial and aspatial); types of maps being produced; and types of mapping tasks in which data analysts would like to receive training.

### Data analysts

For data analysts, we provided training in using EYEMAP, access to the EYEMAP software throughout phase two, software technical assistance as required, as well as ongoing support for questions/issues related to data sources, mapping principals, and so forth. As our project unfolded, it became apparent that some data analysts were using mapping software other than EYEMAP (*e.g.*, MapInfo, Arc Map, and Microsoft MapPoint), so we also provided training relevant to these commercial products. The intervention facilitator delivering these interventions (MZ) is a trained geographer with a strong background in geographic information systems (GIS) and has used MapInfo, Arc/GIS and other GIS packages extensively.

### Managers

For the managers, we provided a series of visits to help train them to interpret spatial data and use it to support local decision making. While it was originally envisioned that these visits would be delivered one-on-one (to managers only), all the managers insisted that their data analysts also participate. At the end of each intervention visit, participants were asked what kind of information they would like to have shared in subsequent visits to ensure that our interventions were tailored to their personal and local decision making needs.

Specifically, the visits with the data analyst/manager dyads covered the following topics:

1. Visit one (GIS basics): Visit one included a tutorial on the basics of GIS. We addressed basic components of geographic data in order to ensure all participants would understand how geographic data representation models are used to represent points, lines, and area surfaces. We discussed the use of symbology, scale, and georeferencing, the method by which one links a geographic location in the real world to a digital map representation through the use of coordinate systems (*i.e.*, longitude and latitude).

2. Visit two (principles of making and interpreting maps): At visit two we delivered further tutorials on the basic principles of map making and the interpretation of geographic data such as density surfaces that illustrate the varying concentration of values within a region and illustrate hotspots and combinatorial surfaces (the overlay of more than one surface where the interest is in a combination of values that occur at the some locations), as well as some of the pitfalls of uncertainty. We also discussed the importance of knowing the source and reliability of the data collected, the scale of analysis (*i.e.*, the representative fraction of its meaning to map uncertainty), the accuracy of the data, and finally, how to avoid committing the ecological fallacy (*i.e.*, attributing characteristics of an area to individuals residing in the area [21]).

3. Visit three (map classification and continued barriers assessment): Having provided general spatial literacy training in the first two visits, visit three served a dual purpose: first, to provide training in one complex issue of data management that all groups would encounter, the classification of area maps (Choropleth); and second, to address the unique needs of each dyad group in order to further reduce barriers to adoption.

4. Visit four (self-assessment tool): The final visit then focused on the use of maps for decision making. Specifically, the purpose of this session was to stimulate a discussion between the manager and the data analyst about their individual and organizational needs around mapping and maps, and then make any system barriers to using local data and maps more transparent for both parties. This approach has been successfully used to promote evidence-based decision making in other contexts [22]. Prior to the visit, the manager and the data analyst were asked to fill out a modified self-assessment tool called *Is Research Working for You? *developed by the Canadian Health Services Research Foundation (CHSRF). The tool asks questions grouped into four main domains: Acquire: can your organization find and obtain the research findings it needs? Assess: can your organization assess research findings to ensure they are reliable, relevant, and applicable to you? Adapt: can your organization present the research to decision makers in a useful way? Apply: are there skills, structures, processes, and a culture in your organization to promote and use research findings in decision making? The comparison of scored items provided a useful starting point for stimulating discussion about the given organization's capacity to use research findings to inform decision making [22].

### Data collection

Phase two data collection took place between September 2006 and March 2009, and involved field notes stemming from manager visits and dyad training sessions, email exchanges between the research team and participants (regardless of who initiated contact), and exit focus groups that were recorded and transcribed verbatim for analysis. As visits three and four were more interactive, these were also taped and transcribed verbatim for analysis. The final exit focus groups occurred in February 2009. Following a brief overview and recap of project findings to date, managers and data analysts were interviewed separately because the nature of the questions were different for the two groups. Managers were more able to comment on how maps were used for decision-making purposes, other contextual factors and issues involving Ministry interactions, whereas data analysts could address more technical issues around the creation of maps and how their maps were received by their managers. Those managers and data analysts that could not attend the in person focus group were interviewed by telephone. Table 1 provides a summary description of interventions delivered and associated data collection techniques used.

**Table 1:** Summary description of delivered interventions and data collection with participants

INNOVATION: Using maps for decision-making purposes			
Target participant	Interventions: series of training/education support	Data collection specific to intervention	Data collection methods consistent across all intervention visits
Data analysts	EYEMAP Software	Participatory design process (see [10]).	
	Market GIS software specific training on individual basis as required	Intervention researcher evaluation of data analyst map creation (done across all visits/interactions).	Field notes from all visits and interactions with participants written up immediately following visit (individually with data analysts and visits with manager/data analyst dyad pairs).
Data analysts and managers	Visit one: GIS basics		
	Visit two: Principles of making and general interpretation of maps		Audio recording transcripts of all visits with participants.
		Individual telephone interviews with managers and data analysts prior to visit three as continued barrier assessment and guide to tailoring visit three.	Email exchanges between participant dyads and research team.
	Visit three: Map classification and interpretation	Dialogue between manager and intervention researcher about interpreting a specific map consisting of mock data.	Individual interviews (telephone and in person).
	Visit four: Self-assessment Tool	Dialogue between manager/data analyst and intervention researcher about respective responses with more detailed probing around key issues.	Focus group exit interviews (in person); individual interviews with participants that could not attend exit focus group (by telephone).

### Data analysis

Our approach to analysis was guided by several principles in qualitative inquiry: data triangulation, checking for consistency in interpretation across transcripts, peer debriefing sessions to seek out alternative explanations/interpretations to the data, and a process of verifying interpretations with participants through 'member-checking' [23-28]. The combination of the different data sources (email exchanges, exit focus groups, and individual interviews) enabled data to be triangulated to confirm interpretations arising from the data. Field notes and interview transcripts were imported into NVivo8 for analysis.

### Data coding

Data were coded by one coder (EC) to ensure consistency in interpretation of text, but the coding categories were developed collaboratively between one research team member (SMD) and the coder. The coding template was guided by elements important in the OMRU for the interventions (challenges/barriers, satisfaction/facilitators, initial and sustained use/adoption, outcomes) in addition to the other domain areas (the innovation, potential adopters, and the practice environment). The coding categories were read by two other team members (SMD, AK) to ensure consistency across transcripts.

### Data verification

Emerging patterns in the data were discussed and any discrepancies were debated, challenged, and resolved at a peer debriefing session at a final team meeting (all). Moreover, a summary report outlining some of the key findings emerging from the project was developed to share with Ministry stakeholders. This summary report was first shared with participants to ensure: accuracy of content and interpretation; protection of participant privacy and confidentiality; and to identify if anything important to participants had been missed. In this way, our project analysis underwent a further process of verification through participant feedback/member checking.

### Measures of map creation

The intervention visits and corresponding field notes written by those delivering the intervention (either a research assistant and co-investigator or a research assistant alone) represented another source of data. In particular, research team members used these data to provide a team assessment of map creation by data analysts. Simple categories were devised to assess map use throughout the research project: 1-none (no map use); 2-external (map use derived from an outside source); 3-limited (in-house map production/limited use in the form of mapping locations of services and simple visualization); 4- intermediate (in-house map production/average use in the form of the exploration of census data and locally collected data); 5- advanced (in-house map production/good understanding of spatial relationships and the creation of meaningful new information by data manipulation).

## Results

### Mapping innovation and interventions

Nine dyads participated at the start of the study; as the study progressed, changes in staff turnover were addressed through tailored modifications to the interventions (*e.g.*, 'catch-up' sessions to bring the individual up to speed). As to be expected, participants were involved to varying degrees throughout the duration of the project due to other commitments (see Table 2). Analysts consistently attended more sessions than managers in each dyad given that they participated in interventions tailored for analysts only, as well those tailored for managers (at the request of managers). This turned out to be a strength as it helped facilitate manager learning during these sessions. Participants who chose not to continue to be involved through the full duration of project cited their primary reasoning for this as being staff changeover and position abeyance, access to commercial mapping software, as well as concern about the ongoing relevance of the project to their organization. With respect to this latter point, while the project was tailored as best as possible, some organizations did feel that they had sufficient mapping experience, or, in some cases, maps/mapping were not sufficiently valued activities, to want to remain in the project. Six dyads participated until the end of the study, representing a completion rate of 67%.

**Table 2:** Dose of interventions received by OEYC dyads

Data Analyst/Manager Dyads	Manager Assessment phase 1	EYEMAP Software	EYEMAP Training 1 June 2006	EYEMAP Training 2 March 2007	Visit 1 Nov 2006	Visit 2 July 2007	Post Visit 2 Data Analyst Assessment	Post Visit 2 Manager Assessment	Visit 3 - Aug 2008	Visit 4 - Nov 2008	Exit Assessment Jan-Mar 2009	Ratio (%) of visits to interventions received
A (Data Analyst)		**x**		**x**	**x**		x		**x**	**x**	x	5/7 (71%)
A (Manager)	x				**x**			x	**x**	**x**	x	3/7 (42%)

B^1 ^(Data Analyst)		**x**	**x**	**x**	**x**	**x**						5/7 (71%)
B^1 ^(Manager)					**x**	**x**						2/7 (28%)

C^2 ^(Data Analyst)		**x**	**x**		**x**		x		**x**	**x**	x	5/7 (71%)
C^2 ^(Manager)					**x**			X	**x**	**x**	x	3/7 (42%)

D (Data Analyst)		**x**	**x**				x		**x**			3/7 (42%)
D (Manager)	x							X	**x**		x	1/7 (14%)

E (Data Analyst)		**x**		**x**	**x**	**x**	x		**x**	**x**		6/7 (85%)
E (Manager)	x				**x**	**x**		X	**x**	**x**	x	4/7 (57%)

F (Data Analyst)		**x**	**x**	**x**	**x**	**x**	x		**x**	**x**	x	7/7 (100%)
F (Manager)	x				**x**	**x**			**x**	**x**		4/7 (57%)

G (Data Analyst)		**x**	**x**	**x**	**x**	**x**	x		**x**			6/7 (85%)
G (Manager)	x				**x**	**x**		X	**x**			7/7 (100%)

H^3 ^(Data Analyst)		**x**	**x**	**x**	**x**	**x**	x		**x**	**x**	x	7/7 (100%)
H^3 ^(Manager)	x				**x**	**x**				**x**		3/7 (42%)

I (Data Analyst)		**x**		**x**		**x**	x		**x**	**x**	x	5/7 (71%)
I (Manager)						**x**			**x**	**x**	x	3/7 (42%)

Notes:

^1^B dropped out of the project after visit 2

^2^C was in the process of staff changeover and did not have a Data Analyst during visit two.

^3^H was in the process of staff changeover and did not have a Manger during visit three

### Map creation

**Figure 1:**
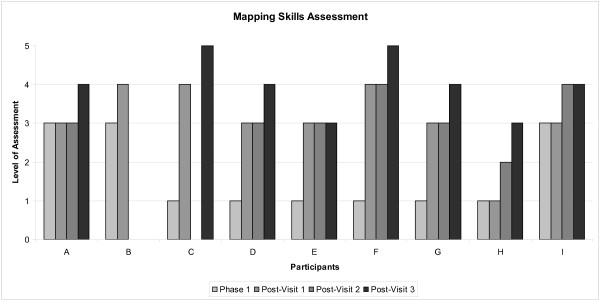
**Evaluation of each data analyst's ability to create maps**. It was noted through the analysis of data collected at visit two, which included the advanced GIS tutorial that most of the groups had improved in their map use. By visit three, four analysts moved from level two up to level three, four analysts moved from level three to level four and two analysts from level four to level five. Note: Data analyst B dropped out of the study after visit one. Data analyst C experienced a change in data analyst following visit one. There was no data analyst during visit two, but a new data analyst was hired before Visit three.

The phase one (Summer 2006) assessment of data analyst's ability to create maps demonstrated considerable variation across sites (Figure 1)--in total nine analysts were assessed and categorized according to their skill level. Six analysts were at categorized as level one (no map use); one analyst at level two (external); three analysts at level three (limited) (see Figure 1). By phase two, visit one, when the introduction to GIS tutorial was presented, with one exception, all of the data analysts that initially did not have access to software other than EYEMAP had acquired commercial software and received some form of training, raising their assessments to a level three or higher. The single analyst that still did not have access to GIS software other than EYEMAP had begun to receive maps from an outside source (level two). Change is reflected in the values from the assessment after visit three, where one analyst moved from level two up to level three, two analysts moved from level three to level four, one analyst from level four to level five and two analysts exhibited no change in skill level.

Although there was a marked change amongst the participants over the course of the project in their personal comfort with mapping, they still encountered questions in their work environment about what maps could actually do and what constituted spatial data. Because the overall level of spatial literacy remained low amongst the individuals with whom the data analysts worked, analysts often felt limited by the information that was requested from them:

'I think most people still think of maps in static terms, this is a map, but this map comes from data and I can draw you a different map that shows something else from these data, and maps, and geographers are using maps in the dynamic way and their presentations ... as they [maps] go ... I think ... we're a long way away from thinking in those terms. ... I keep being asked for a map, what I'm asked for is a piece of paper this size ... that shows some information, I'm not being asked to use geographic information that's a sophisticated way of being asked, its this piece of paper and that's, that's a limiting factor.' (Data Analyst)

Most of the data analysts did not have formal training as geographers. The creation and use of maps as a tool for data analysis were recent parts of their duties. Throughout the course of the project, we saw individual awareness about mapping change. As individuals saw maps as an important tool, individuals seemed to be working towards gaining a better understanding of how and when to use maps, (and how to create maps with limited access to data sets, such as postal code boundary files). The recognition that maps are an important tool is clearly evident to both managers and data analysts alike:

'I'm mapping probably almost, well almost every project, I'd do some kind of map, whether its postal codes, census, earlier services, locations ... I would say its becoming more frequent because we do have access to the software now ... it's going to be integrated in what we do.' (Data Analyst)

The additional tailored tutorials that we provided data analysts following the intervention visits with them and their managers might have contributed to an increase in map creation. We responded specifically to their articulated needs, providing data analysts with tutorials on adding new data to a map, deriving geographic coordinates from online sources, standardizing census data for population density, and calculating net residential density. Moreover, some analysts' data included population numbers for areas that contained adjacent pockets of residential and commercial zoning which skewed population density calculations. We provided support for this issue by providing guidelines to recalculate population density by excluding commercial areas known to contain no residential housing. It is unclear, however, from our study data itself, exactly how much of a role our tailored interventions on these issues may have increased map creation among data analysts.

### Map use to support decisions

One of the goals of our intervention with participants was to encourage a common geographic language between data analysts and managers. Visits one and two were designed to provide managers with a background in some key mapping concepts to better enable them to know the kinds of questions that they could ask of their data analysts when examining locally relevant data. Visit three was designed to increase this skill by reviewing sample maps with managers and discussing map interpretation.

Some participants reported that maps played an important role in the decision-making process regarding the location of services:

'So we, we provided some, some mapping data, we've taken each of the neighbourhoods and then we can start, we can look at what, we can map by social riskiness, they can see where the highest areas of risk are. We've also taken it individually by overlay population, density with certain census data ...like low income stats...the best places in the city for this clinic.' (Data Analyst)

Many participants noted that maps had the advantage of being able to synthesize different types of data together for visual analysis. More often than not, managers made decisions about gaps in services (*i.e.*, map-and-gap analysis) in conjunction with their community partners. In other words, map use went beyond the organization--maps supported the planning of programs community-wide:

'I would also use the maps for decision making because we are a small municipality so we work in partnership with our community partners, so these kinds of things help us all to determine where we might need a [x] office ... the maps become rather valuable in those kinds of decisions.' (Manager).

'I think probably everybody's looking for something different within a map. For my staff, it's probably going to be, oh, perhaps looking at where the families are coming from and they probably are going to identify because they've been in conversations with the family. For me, it would be looking at where we could put new programming. For maybe my board, it's going to be looking at the general view point of the number of families that have been in there.' (Manager)

Maps also took on a program evaluation function. Often, programs were provided by community partners, and as one manager participant stated:

'And so, that's where you get a lot of interesting information, because particularly in the community locations, so I just received the maps, so now these will go to the staff and say, okay, here's your community program, this is what the map's telling us, and then hopefully they're going to bring back information [to their home agency] ... [Such as] wow, we don't have people coming right, that are right next door to us, how can we look at that ... So I'm hoping that that type of information will be generated by them without me having to say, this is it.'

In the example above, the manager is hoping that the map will provide enough evaluative feedback to the community partner that the organization will be motivated into action for improvement without having an explicit discussion about outcomes or performance.

Maps were often used to confirm the tacit knowledge that managers held about their communities:

'...Usually, people connect with information better I think that way than seeing it on a chart, but you always have a feeling that something, like for me with programming, you have a feeling that some things are working in some areas and perhaps not working in others, ... really supports I guess your gut feelings ...' (Manager)

'...Say, for instance, I know that in the north end of our county that's where the EDI scores were a little lower, not an awful lot, but a little lower than the rest of the county or than the provincial norm, so that's were I would you know, if you [asked me where] to put in a full day early learning, where would you say it should go? I would probably say either in the central part of our county or in the north part of our county, or in a small pocket in the south end, like I know that! ... You know, in rural communities we know, we know just because we know um, where programs are best situated.' (Manager)

This use of maps might correspond to Weiss' symbolic use, which refers to the use of research and information to support a decision already made.

It seemed that mapping was approached in a realistic way in that the potential disadvantages of maps were understood by some, perhaps due to the project intervention visits. For example, a few participants mentioned how a large geographic area with a low EDI score might seem quite prominent on a visual display (*i.e.*, the map), and this could be misleading. In one instance, the participant described how a map was 'too influential' and 'too powerful.' In this case the map led to the placement of services in some seemingly low EDI score areas. Upon further inspection, however, it became clear that the proper inferences had not been made due to the low population density in these areas.

In a few cases, data analysts and managers expressed their intentions to use maps in the future, indirectly indicating their general support for maps as a decision-making tool. In other cases, there was some evidence that the use of maps was on its way to becoming an institutionalized practice:

'I think it's becoming part of what we do now and how we relate to data ... it's part of how we're using information now to, to make decisions, ... so definitely I would say its definitely being used and will be used much more.' (Data Analyst)

'...like some community partners won't even make decisions until they, they've requested a map and view it first, like they've, they understand that mapping is available, that it's a great tool, so there's some instances where they basically won't make a decision ... and, in other instances, they just generally like to know and to feel good about their programming decisions and using mapping as a tool to sort of support that.' (Data Analyst)

### Impact of mapping and maps

It is difficult to determine the impact of mapping and maps in this project. While OMRU provides guidance for measuring outcomes and impacts, we were unable to collect external data that could have provided a measurement of impact. While we made attempts to obtain external reports (*e.g.*, annual reports, community or meeting documents) produced by OEYCs to independently evaluate against participant self-report, these external reports were not made available to the research team in a comprehensive fashion to be functionally used. Further, EYEMAP's use by analysts was generally low and the application itself likely had little overall impact on mapping. While there was usually an increased level of usage immediately following training sessions, this was found to quickly drop off. As well, EYEMAP was generally only explored by analysts who were intermediate- or advanced-level users. Nonetheless, data analysts did increase their capacity to make maps over the course of the project (see Figure 1), and there was evidence that managers spoke about the importance of maps to support decision making more strongly at the end of the project. Determining how much of this impact was associated with this particular intervention project, or how sustainable this change will be over the long term remains unknown.

## Discussion

While there was more mapping and use of maps following our KT intervention, there is little evidence to suggest that this increase was substantial. We have a number of hypotheses for why there was not greater mapping/use. These hypotheses relate to the principles of OMRU: the innovation itself, the adopters, the environment, the KT intervention, and outcome measurement issues.

### The innovation

There were some issues that arose with the mapping innovation, in particular the software/technology development. Phase one discussions with participants indicated that there was a need for a more user-friendly and web-based mapping software, to which our project responded with the development of EYEMAP. There were two features of EYEMAP that participants in phase one expressed considerable interest in, and that we believe are the hallmark features of EYEMAP: the spatial data sharing and map interoperability features (*i.e.*, a map created by one OEYC can be viewed and modified by other OYECs even if they do not have the original source mapping software or data). Ease of spatial data sharing was one of the original needs identified by data analysts. However, while this feature was at the forefront of our development, despite its availability, combined with the full range of other standard GIS features, EYEMAP was not widely adopted by participants. We feel that perhaps these features arrived too soon in the project for their utility to be realized. Based on our observations and data, maps were not being used enough initially for sharing to be of practical importance. Mapping is in its infancy in this sector, and consequently the sharing and interoperability features were undervalued. It could be expected that such features would have been more highly valued if users had initially been more advanced.

Moreover, while a participatory software design was used, with the benefit of hindsight, it increased the length of the EYEMAP development cycle to over a full year. As such, some of the concepts and capabilities the data analysts needed early on were found through other avenues. For example, data analysts wanted a geocoding functionality (*i.e.*, turning street addresses into latitude and longitude coordinates to be mapped). During the EYEMAP development periods, free web-based applications became available (*e.g.*, Statistics Canada released free street network files that promoted new free online geocoding services available to anyone). This signals that web-based interventions, like ours, require faster turnaround than what our participatory process was able to deliver. With the availability of recent and stable free and open source GIS software [29-31], a combination that was unavailable at the start of our project, future interventions should work within existing software capabilities and focus more on specific training in GIS to support decision making processes.

Additional reasons to explain why there were challenges with the innovation relate to factors described below in the contextual environment in which these OEYCs operated (*i.e.*, related to the financial climate and data sharing agreements).

### The adopters

While there was considerable initial interest in the project, and a reported recognition of the potential utility of maps and mapping software to support decision making at the local level, long term project buy-in was difficult to maintain. Ours was a long-running project, having started in early 2004 with preliminary data collection. At that time there were no web mapping systems like EYEMAP in the public domain; for example, Google Earth^® ^was not released, and Google^® ^maps and similar web mapping software were in their infancy, and open-source GIS were immature and not user-friendly. EYEMAP was innovative and bridged a needed gap between the required advanced mapping functions for decision making and the required needs of data analyst novice users, thus filling a niche for mapping and analysis. However, sustaining interest and excitement over a long period of time is difficult in the face of rapidly changing and attractive project-external mapping technology. Nevertheless, data analysts demonstrated an increased capacity in creating maps (using other software), and managers (not to mention their communities at large) confirmed through qualitative self-reports an increased use of maps for supporting decision making. While this project may have contributed to this finding, there were a number of other things happening concurrently as mentioned that may have also contributed to the increase in map generation and use that relate to the environment.

### The environment

Several external factors likely created a general environment that was conducive to mapping and map use that supported our project intervention. One of the biggest contextual influences over this period was the active support for the use of Early Development Indicator (EDI) data provided by the Offord Centre for Child Studies in Hamilton, Ontario. The Offord Centre would return to each OEYC its own EDI data that was cleaned, anonymized, and geo-referenced to the postal code level. The Offord Centre also provided data analysts with some basic maps if the OEYC did not have any capacity to create its own. This access to EDI data, in a format that had not been available to data analysts before, was novel. Prior to the Offord Centre, EDI data tended to be spatially referenced by the postal code of children's schools, as opposed to children's homes. This meant that it was practically impossible for OEYCs to examine the relationships between EDI scores and neighbourhood access to programs and services (see [11] for some phase one examples of this problem). Thus, it is probable that the identified increase in mapping activities is at least in part attributable to a greater access to appropriately georeferenced, planning relevant data.

At the same time, the environment impeded the use of maps and mapping both in terms of data access issues and the financial climate. There was a substantial disparity in data access noted by our participants depending on whether they were in a predominantly urban resource-richer area (*i.e.*, data access, training opportunities, *et al*.) compared to a rural resource-poorer area. Moreover, data analysts often interacted with other public health professionals (*e.g.*, epidemiologists) that had access to census and other data that the OEYC itself did not have. To illustrate, participants indicated that each OEYC is responsible for purchasing its own census data. Participants also indicated during the project early phase that they are responsible for paying for the Postal Code Boundary Files that permit analysts to match neighbourhoods and dissemination areas in their region with postal code boundaries; some of these features are now freely available. Yet, these same data analysts could not use the data that other public health professionals had access to because another provincial ministry paid for that data. One of the data-sharing regulations of consortium agreements is that such data cannot be shared outside participating members. A number of our participating dyads wondered why the province does not enter into larger data sharing agreements for common data sets that a number of departments and Ministries rely upon for program planning and service delivery.

Another major barrier was the poor financial situation of all OEYCs. Since the inception of OEYCs in the province in 2002, there has been no increase in funding. OEYC managers have been struggling to continue providing more and more services with fewer dollars. This financial climate contributed to a less supportive environment for the added time and human resources needed to produce and use maps. In the words of one manager, 'you can do as many fancy maps as you want, we're still not going to get any more money' (visit four meeting).

### The KT intervention

Another factor that may have affected the extent to which maps and mapping was adopted in decision making is the dose of the intervention (*i.e.*, number of visits, length of visits, and quality of visits). There is evidence from some of our discussions with data analysts and managers that some participants did not fully understand what the project was offering through its intervention visits. For example, one data analyst commented that they did not map much because they lacked access to GIS software--despite the fact that EYEMAP provided many features of market-based GIS programs--until the end of our project when they acquired MapInfo. Similarly, one manager was excited to learn, at the end of our project, that training would be provided on spatial/map interpretation despite the fact that they participated in every visit we provided. These findings suggest that the 'dose' was, at least in some cases, inadequate and that further interventions with added preamble with participants are required.

Lastly, as is often the case with implementation studies [32-36], we have no way to measure the true impact of our KT interventions. If done too soon following the intervention, the effect may not be picked up, and by waiting too long, recall bias could interfere with providing an accurate reflection of effect.

Thus, there were barriers as well as facilitators that represented the context within which this study was immersed. Participants demonstrated a growing engagement with mapping software. Perhaps more importantly, maps were being used in decision-making forums as a way to allocate resources, confirm tacit knowledge about community needs, make financially-sensitive decisions more transparent, evaluate programs, and work with community partners.

### Study limitations

Our project relies on self-reported assessments on the part of participants which are appropriate to assessing the process of intervention and implementation, as opposed to having objective evaluative outcome measures that would be ideally available to fully assess the specific features of the OMRU. While there were only six-dyads (12 participants) this was offset by the depth of the data and our ability to triangulate against these multiple data sources. As such, we have opted for a 'weight of evidence' approach to evaluating the different pieces of collected data over the length of the project: participatory meetings with participants, emails between participants and project staff/researchers, discussions and conversations during the delivery of intervention visits, telephone and in-person interviews, *et al*. The interdisciplinary nature of the research team allowed us to minimize study limitations in the analysis of self-reported interview and other observational data collected in this project. Given the resulting diversity in investigator backgrounds and professional training, the investigators, through peer-debriefing sessions, brought different perspectives to the data analysis that minimized the chance of interpretive bias from a single perspective and expertise. The multiple data sources that we had to draw from (observation notes, interactions with participants, interviews, *et al*.) provided the research team with a means to triangulate emerging interpretations and project analysis through different means. Moreover, participants had opportunities to provide feedback to emerging interpretations throughout the project, as well as through the process of vetting a project summary report to be shared with the provincial Ministry of Child and Youth Services.

## Summary

This study provides some initial insights in doing an integrated KT project with research producers and research users working in the same organization, as well as some insights in using maps as a KT tool. In terms of mapping, participants indicated that in the right contexts, maps can be very useful KT tools for turning data-rich environments into information-rich environments where such information can be used to help support decision making. While our participants made considerable advances in their capacity to do mapping, there is still a significant learning curve to overcome in terms of communicating the benefits of maps as a decision-support system.

One of the strongest facilitators in this project's context that was a pleasant surprise to us was how closely the data analysts and managers work together, and how collaborative a community the data analysts are in supporting each other. For example, managers insisted that their data analysts be present during 'manager training sessions.' This helped to increase learning among managers as data analysts were often able to provide local context and examples for how their local data could help answer different questions through mapping. Moreover, in many cases, data analysts worked with a considerable amount of autonomy in making decisions about what data they would look at, how they would analyze them, and what findings they would present to their managers. They essentially comprise a community of practice. That said, the loss of one key champion among the data analysts from the community of practice (due to job transfer and position abeyance) created a vacuum that left a substantial variability in the remaining skill set among the data analysts.

More research is required to understand how mapping software and maps can support evidence-based decision making in public health program planning. Future work should focus on minimizing the innovation and adopter barriers identified here. For example, innovation barriers could be removed by introducing off-the-shelf commercial mapping or mature and stable open-source software [30-32] that are now relatively common as compared to 2004, and adopter barriers could be limited by shortening the intervention period. Further work also needs to be done to better understand how maps may support evidence-based decision making in different public health contexts, such at the level of Ontario's Public Health Units or Local Health Integration Networks. Doing so would also help us to understand the relative role of environmental barriers to the adoption of maps and mapping in decision making. Lastly, the challenge of capturing impact and the sustainability of such changes in behaviour from intervention studies remains a challenge for KT researchers; one that can only be somewhat offset by the use of a strong theoretical model guiding the research.

## Competing interests

The authors declare that they have no competing interests.

## Authors' contributions

SMD and JM conceived, SMD, AK and IDG designed, and SMD and AK implemented the project. JM took the lead on the design of the software and MS provided expertise on the GIS components. SMD, AK, EC, and MZ were involved in the collection of the data, and EC, SMD, and AK were involved in the analysis of data. MZ designed the tutorials used during the first three visits delivered to participants with substantive feedback from all team members. All team members contributed to the interpretation of findings. IDG provided guidance with the structure of the manuscript as per the conceptual framework used in this project, developed in part by IDG. While SMD took the lead in drafting the manuscript, AK, IDG, EJC, and MS provided substantial feedback and written contributions to the draft manuscript, and EC and MZ contributed to the writing of select sections. All authors read and approved the final manuscript.
